# Planar chemical reaction systems with algebraic and non-algebraic limit cycles

**DOI:** 10.1007/s00285-025-02221-0

**Published:** 2025-05-22

**Authors:** Gheorghe Craciun, Radek Erban

**Affiliations:** 1 Department of Mathematics and Department of Biomolecular Chemistry, University of Wisconsin—Madison, 480 Lincoln Dr, Madison, WI 53706-1388 USA; 2 Mathematical Institute, University of Oxford, Radcliffe Observatory Quarter, Woodstock Road, Oxford, OX2 6GG UK

**Keywords:** Chemical reaction networks, Limit cycles, Hilbert number, Algebraic limit cycles, 34C07, 14P05, 80A30, 37N25

## Abstract

The Hilbert number *H*(*n*) is defined as the maximum number of limit cycles of a planar autonomous system of ordinary differential equations (ODEs) with right-hand sides containing polynomials of degree at most $n \in {{\mathbb {N}}}$. The dynamics of chemical reaction systems with two chemical species can be (under mass-action kinetics) described by such planar autonomous ODEs, where *n* is equal to the maximum order of the chemical reactions in the system. Analogues of the Hilbert number *H*(*n*) for three different classes of chemical reaction systems are investigated: (i) chemical systems with reactions up to the *n*-th order; (ii) systems with up to *n*-molecular chemical reactions; and (iii) weakly reversible chemical reaction networks. In each case (i), (ii) and (iii), the question on the number of limit cycles is considered. Lower bounds on the modified Hilbert numbers are provided for both algebraic and non-algebraic limit cycles. Furthermore, given a general algebraic curve $h(x,y)=0$ of degree $n_h \in {{\mathbb {N}}}$ and containing one or more ovals in the positive quadrant, a chemical system is constructed which has the oval(s) as its stable algebraic limit cycle(s). The ODEs describing the dynamics of the constructed chemical system contain polynomials of degree at most $n=2\,n_h+1.$ Considering $n_h \ge 4,$ the algebraic curve $h(x,y)=0$ can contain multiple closed components with the maximum number of ovals given by Harnack’s curve theorem as $1+(n_h-1)(n_h-2)/2$, which is equal to 4 for $n_h=4.$ Algebraic curve $h(x,y)=0$ with $n_h=4$ and the maximum number of four ovals is used to construct a chemical system which has four stable algebraic limit cycles.

## Introduction

The dynamics of chemical reaction networks under mass-action kinetics is inherently connected with the investigation of the dynamics of ordinary differential equations (ODEs) with polynomial right-hand sides (Feinberg 2019; Angeli 2009). In this paper, we consider chemical reaction networks with two chemical species *X* and *Y*. Denoting the time-dependent concentrations of chemical species *X* and *Y* by *x*(*t*) and *y*(*t*), respectively, their time evolution is described by a planar system of ODEs

1.1 $$\begin{aligned} \frac{\text{ d }x}{\text{ d }t}= &  f(x,y) \,, \end{aligned}$$

1.2 $$\begin{aligned} \frac{\text{ d }y}{\text{ d }t}= &  g(x,y) \,, \end{aligned}$$

where *f*(*x*, *y*) and *g*(*x*, *y*) are polynomials. The ODE systems in the form (1.1)–(1.2) have been investigated in detail since the pioneering work of Bendixson (1901), who showed that the most complex long term dynamics one can expect to observe in planar systems are multiple limit cycles. Considering that *f*(*x*, *y*) and *g*(*x*, *y*) are polynomials of degree at most $n \in {{\mathbb {N}}}$, it is interesting to ask how many limit cycles ODEs (1.1)–(1.2) can have. While this question in its full-generality is a part of the yet-unsolved Hilbert’s 16th problem (Christopher and Li 2007), it can be answered when there are additional restrictions imposed on the right-hand sides of the ODEs (Póta 1983; Schuman and Tóth 2003; Gasull and Giacomini 2023). A related question is to find (in some sense minimal) examples of planar polynomial ODE systems (1.1)–(1.2) for low values of *n*, which have a certain number of limit cycles or specific bifurcation structure (Shi 1980; Li et al. 2009; Plesa et al. 2016; Erban et al. 2009).

Chemical reaction networks consisting of reactions with order at most $n \in {{\mathbb {N}}}$ can be described by ODEs in the form (1.1)–(1.2), where *f*(*x*, *y*) and *g*(*x*, *y*) are polynomials of degree at most *n*, which have some further restrictions on their coefficients. In Sect. 2, we define three important classes of chemical reactions networks: (i) set ${{\mathbb {S}}}_n$ consisting of reactions of at most *n*-th order; (ii) set ${{\mathbb {M}}}_n$ consisting of reactions which are at most *n*-molecular; and (iii) set ${{\mathbb {W}}}_{\!n}$ consisting of the networks in ${{\mathbb {M}}}_n$ which are weakly reversible (Craciun et al. 2020). Our main question is to understand the existence of limit cycles in sets ${{\mathbb {S}}}_n$, ${{\mathbb {M}}}_n$ and ${{\mathbb {W}}}_{\!n}$, either by finding relatively simple chemical networks with a certain number of limit cycles, or by proving that certain numbers and configurations of the limit cycles cannot exist. Denoting the maximum number of limit cycles in sets ${{\mathbb {S}}}_n$, ${{\mathbb {M}}}_n$ and ${\mathbb W}_{\!n}$ by *S*(*n*), *M*(*n*) and $W{\hspace{-0.85358pt}}(n)$, respectively, we study the counterparts of the Hilbert number *H*(*n*) in the chemical reaction network theory (Erban and Kang 2023). In Sect. 3, we provide estimates on the values of *S*(*n*), *M*(*n*) and $W{\hspace{-0.85358pt}}(n)$ for small values of *n* and in the asymptotic limit $n \rightarrow \infty .$

An important subset of limit cycles in planar polynomial ODE systems  (1.1)–(1.2) are algebraic limit cycles (Chavarriga et al. 2004; Gasull and Giacomini 2023). An algebraic limit cycle is not only a closed isolated solution of the ODE system, but it can also be represented as a closed component of an algebraic curve $h(x,y)=0$, where *h* is a polynomial. The simplest examples of algebraic curves include circles and ellipses. Some chemical systems that have an ‘exactly evaluable’ (algebraic) limit cycle given as an ellipse were analyzed by Escher (1979, 1980a, 1980b). In Sect. 4, we focus on constructing chemical systems with *algebraic* limit cycles. In particular, we further specialize the numbers of limit cycles *S*(*n*), *M*(*n*) and $W{\hspace{-0.85358pt}}(n)$ in sets ${{\mathbb {S}}}_n$, ${{\mathbb {M}}}_n$ and ${{\mathbb {W}}}_{\!n}$ to quantities giving the maximum number of algebraic limit cycles, denoting them by $S^a(n)$, $M^a(n)$ and $W^a(n)$. If the degree of polynomial *h* is $n_h=2$ or $n_h=3$, then the algebraic curve $h(x,y)=0$ contains at most one closed oval (a connected component diffeomorphic to a circle). If $n_h=4,$ then Harnack’s curve theorem implies that the maximum number of connected components is 4. In Sect. 5.1, we study an algebraic curve $h(x,y)=0$ of degree $n_h=4$ which has the maximum number of ovals, 4, for some parameter values. We construct a chemical system which has all four ovals as stable limit cycles. We conclude with the discussion of our results and the literature in Sect. 6.

## Chemical reaction networks in sets ${{\mathbb {S}}}_n$, ${{\mathbb {M}}}_n$ and ${{\mathbb {W}}}_{\!n}$

We consider chemical reaction networks with two chemical species *X* and *Y* which are subject to $m \in {{\mathbb {N}}}$ chemical reactions

2.1 $$\begin{aligned} \alpha _i X \, + \, \beta _i Y \; \mathop {\longrightarrow }^{k_i} \; \gamma _i X \, + \, \delta _i Y \,, \qquad \text{ for } \quad i = 1, 2, \dots , m, \end{aligned}$$

where $\alpha _i \in {{\mathbb {N}}}_0,$ $\beta _i \in {{\mathbb {N}}}_0$, $\gamma _i \in {{\mathbb {N}}}_0$ and $\delta _i \in {{\mathbb {N}}}_0$ are nonnegative integers, called *stoichiometric coefficients*, and $k_i$ is the corresponding reaction *rate constant* which has physical units of [time]$^{-1}$[concentration]$^{1-\alpha _i-\beta _i}$. However, in what follows, we assume that all chemical models have been non-dimensionalized, i.e. the rate constant $k_i$ is assumed to be a positive real number for $i=1,2,\dots ,m$. Moreover, to avoid some degenerate cases, we assume that the same four-tuple $(\alpha _i, \beta _i,\gamma _i,\delta _i)$ does not occur more than once in the set of *m* chemical reactions (2.1) and we have $(\alpha _i, \beta _i) \ne (\gamma _i,\delta _i)$, i.e. in each reaction step at least one of the two chemical species *X* and *Y* changes. To get non-trivial planar systems, we also assume that both species take part in at least one reaction in the set of *m* chemical reactions (2.1). We then define the *order* of the chemical reaction network (2.1) by

2.2 $$\begin{aligned} n \, = \, \max _{i=1,2,\dots ,m} (\alpha _i+\beta _i) \,, \end{aligned}$$

i.e. the chemical reaction network (2.1) is of the *n*-th order, if all individual reactions are of at most *n*-th order, where the order of an individual reaction is given as $(\alpha _i+\beta _i)$. Assuming mass-action kinetics, the time evolution of the chemical reaction network (2.1) is given by the reaction rate equations which is a planar polynomial ODE system in the following form (Feinberg 2019; Erban and Chapman 2020)

2.3 $$\begin{aligned} \frac{\text{ d }x}{\text{ d }t}= &  \sum _{i=1}^m k_i \, (\gamma _i-\alpha _i) \, x^{\alpha _i} \, y^{\beta _i} \,, \end{aligned}$$

2.4 $$\begin{aligned} \frac{\text{ d }y}{\text{ d }t}= &  \sum _{i=1}^m k_i \, (\delta _i-\beta _i) \, x^{\alpha _i} \, y^{\beta _i} \,. \end{aligned}$$

This ODE system is of the form (1.1)–(1.2), where *f*(*x*, *y*) and *g*(*x*, *y*) are polynomials of degree at most *n*, where *n* is the order of the chemical reaction network given by (2.2).

**Example.** The Lotka-Volterra system can be written as a chemical reaction network in the form (2.1) with $m=3$ chemical reactions and stochiometric coefficients

$$\begin{aligned} &  (\alpha _1,\beta _1,\gamma _1,\delta _1) = (1,0,2,0), \qquad (\alpha _2,\beta _2,\gamma _2,\delta _2) = (1,1,0,2), \\ &  (\alpha _3,\beta _3,\gamma _3,\delta _3) = (0,1,0,0), \end{aligned}$$

i.e. the chemical reactions are

2.5 $$\begin{aligned} X \; \mathop {\longrightarrow }^{k_1} \; 2 X \,, \qquad \qquad X \, + \, Y \; \mathop {\longrightarrow }^{k_2} \; 2 Y \,, \qquad \qquad Y \; \mathop {\longrightarrow }^{k_3} \; \emptyset , \end{aligned}$$

where the reaction rate constants $k_1$, $k_2$ and $k_3$ are positive real numbers. The Lotka-Volterra system (2.5) is given by a chemical reaction network of the second-order, i.e. equation (2.2) gives $n=2$. The corresponding ODE system (2.3)–(2.4) is a planar quadratic ODE system of the form

2.6 $$\begin{aligned} \frac{\text{ d }x}{\text{ d }t} \,= &  \, k_1 \, x \, - \, k_2 \, x \, y \,, \end{aligned}$$

2.7 $$\begin{aligned} \frac{\text{ d }y}{\text{ d }t} \,= &  \, k_2 \, x \, y \, - \, k_3 \, y \,. \end{aligned}$$

### Definition 1

Let $n \in {{\mathbb {N}}}.$ We denote by $\,{{\mathbb {S}}}_n$ the set of chemical networks (2.1) which are of at most *n*-th order, where the order is defined by (2.2).

In what follows we will make a convenient abuse of terminology, and use ${{\mathbb {S}}}_n$ to denote not only the set of the chemical reaction networks described above, but also the set of the corresponding ODEs (2.3)–(2.4), which are planar autonomous ODE systems in the form (1.1)–(1.2), where *f*(*x*, *y*) and *g*(*x*, *y*) are polynomials of degree at most *n*. In general, such polynomials can be expressed in terms of coefficients as

2.8 $$\begin{aligned} f(x,y) \, = \, \sum _{\{i,j \ge 0 \; | \;i+j \le n\}} a_{i,j} \, x^i \, y^j \qquad \text{ and } \qquad g(x,y) \, = \, \sum _{\{i,j \ge 0 \; | \;i+j \le n\}} b_{i,j} \, x^i \, y^j , \end{aligned}$$

where $a_{i,j}$, $b_{i,j}$ are real numbers. The set ${{\mathbb {S}}}_n$ can then be characterized in terms of restrictions on these coefficients, which we state as our next lemma.

### Lemma 1

Consider an ODE system in the form (1.1)–(1.2), with *f* and *g* given by equation (2.8). Then a necessary and sufficient condition for belonging to the set $\,{{\mathbb {S}}}_{n}$ is that the coefficients of *f* and *g* in (2.8) satisfy the following condition

2.9 $$\begin{aligned} a_{0,i} \ge 0 \qquad \text{ and } \qquad b_{i,0} \ge 0 \qquad \text{ for } \quad i=0,1,2,\dots ,n. \end{aligned}$$

### Proof

This is a classical result (Hárs and Tóth 1981). We include a short proof here, since the construction in this proof will be used again later. Consider some term of the form $a_{i,j} \, x^i \, y^j$ that is one of the monomials within *f*(*x*, *y*) in (2.8). In particular, we have $i+j \le n$. We will exhibit a reaction that gives rise to this term $a_{i,j} \, x^i \, y^j$, and no other terms; in other words, this reaction generates the system

$$\begin{aligned} \frac{\text{ d }x}{\text{ d }t} \, = \, a_{i,j} \, x^i \, y^j \qquad \qquad \text{ and } \qquad \qquad \frac{\text{ d }y}{\text{ d }t} \, = \, 0. \end{aligned}$$

Indeed, if $a_{i,j} < 0$ it follows that $i \ge 1$ (because $a_{0,i} \ge 0$), and then this term can be obtained by using the reaction $iX + jY \rightarrow (i-1)X + jY$, if we choose its reaction rate constant to be $k=-a_{i,j}$. Similarly, if $a_{i,j} > 0$, this term can be obtained by using the reaction $iX + jY \rightarrow (i+1)X + jY$, by choosing its reaction rate constant to be $k=a_{i,j}$.

We can proceed similarly for monomials of *g*(*x*, *y*), by using reactions of the from $iX + jY \rightarrow iX + (j-1)Y$ and $iX + jY \rightarrow iX + (j+1)Y$. We conclude that, by using the reaction network which consists of the reactions described above (i.e., one reaction for each monomial of *f*(*x*, *y*), and one reaction for each monomial of *g*(*x*, *y*)), we can obtain the system (1.1)-(1.2).

Conversely, we can see in (2.3)–(2.4) that if $a_i = 0$ then $k_i(\gamma _i - \alpha _i) \ge 0$ and, similarly, if $b_i = 0$ then $k_i(\delta _i - \beta _i) \ge 0$. This implies that any polynomial dynamical system that is generated by a chemical reaction network satisfies the inequalities (2.9). $\square$

The Lotka-Volterra system (2.5) is an example of a chemical reaction network in set ${{\mathbb {S}}}_2$, because every reaction is of at most second-order. Moreover, we observe that not only each reaction in (2.5) has at most two reactants, but it also has at most two products. We will denote the set of such networks as ${{\mathbb {M}}}_2$ and call them *bimolecular* reaction networks. In general, we define the set of *n*-molecular reaction networks ${{\mathbb {M}}}_n$ as follows.

### Definition 2

Let $n \in {{\mathbb {N}}}.$ We denote by $\,{{\mathbb {M}}}_n$ the subset of $\,{{\mathbb {S}}}_n$ which corresponds to chemical reaction networks (2.1), where each chemical reaction has at most *n* reactants and *n* products, i.e.

2.10 $$\begin{aligned} \max _{i=1,2,\dots ,m} \max \big \{ (\alpha _i+\beta _i), (\gamma _i+\delta _i) \big \} \, \le \, n \,. \end{aligned}$$

The set of *n*-molecular reaction networks ${{\mathbb {M}}}_n$ can again be characterized in terms of the coefficients as stated in the next lemma.

### Lemma 2

Consider an ODE system in the form (1.1)–(1.2), with *f* and *g* given by equation (2.8). Then a necessary and sufficient condition for belonging to $\,{{\mathbb {M}}}_{n}$ is to satisfy inequalities (2.9) together with

2.11 $$\begin{aligned} a_{i,n-i} + b_{i,n-i} \le 0 \qquad \quad \text{ for } \quad i=0,1,2,\dots ,n. \end{aligned}$$

### Proof

Consider an ODE system (1.1)–(1.2), with *f* and *g* expressed in coefficients (2.8) that satisfy (2.9) and (2.11). In particular, according to Lemma 1, this system belongs to ${\mathbb S}_n$, which implies that it can be realized by a reaction network where all the reactions are of the form $iX+jY \rightarrow pX+qY$ with $i+j \le n$. We need to show that we can also choose these reactions such that $p+q \le n$.

Note that we know from the proof of Lemma 1 that we can obtain the same monomial terms by using reactions such that $(p,q) = (i \pm 1, j)$ or $(p,q) = (i, j \pm 1)$, and therefore $p+q \le i+j+1$. This gives us the desired conclusion if $i+j \le n-1$. Consider now the case $i+j = n$, i.e. $j=n-i.$ If $i>0$, $n-i>0$ and $a_{i,n-i} \ge b_{i,n-i}$, then we can realize the system

2.12 $$\begin{aligned} \frac{\text{ d }x}{\text{ d }t} \, = \, a_{i,n-i} x^i y^{n-i} \qquad \text{ and } \qquad \frac{\text{ d }y}{\text{ d }t} \, = \, b_{i,n-i} x^i y^{n-i} \end{aligned}$$

by using the following two chemical reactions

$$\begin{aligned} i \, X \, + \, (n-i) \, Y \; \xrightarrow {\;\frac{a_{i,n-i} - b_{i,n-i}}{2}\;} \; (i+1) \, X \, + \, (n-i-1) \,Y \end{aligned}$$

and

$$\begin{aligned} i \, X \, + \, (n-i) \, Y \; \xrightarrow {\;\frac{-a_{i,n-i} - b_{i,n-i}}{2}\;} \; (i-1) \, X \, + \, (n-i-1) \, Y. \end{aligned}$$

Similarly, if $i>0$, $n-i>0$ and $a_{i,n-i} \le b_{i,n-i}$, then we can realize ODE system (2.12) by using two reactions $iX+(n-i)Y \rightarrow (i-1)X+(n-i+1)Y$ and $iX+(n-i)Y \rightarrow (i-1)X+(n-i-1)Y$.

Finally, if $i=0$, then we know that $a_{0,n} \ge 0$, and we can realize ODE system (2.12) by using two reactions $nY \rightarrow X+(n-1)Y$ and $nY \rightarrow (n-1)Y$ with reaction rate constants $a_{0,n}$ and $-a_{0,n} - b_{0,n}$, respectively. The case where $i=n$ is analogous to the case $i=0$. $\square$

**Fig. 1:**
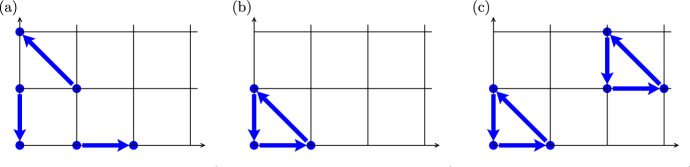
Schematics of planar E-graphs associated with **(a)** Lotka-Volterra chemical system (2.5); **(b)** chemical system (2.13); **(c)** chemical system with six reactions given by (2.13) and (2.16)

The Lotka-Volterra system (2.5) is an example of a chemical reaction network belonging to both ${\mathbb S}_2$ and ${{\mathbb {M}}}_2$. It is described by the conservative dynamical system (2.6)–(2.7), with the conserved quantity $k_2 (x+y) - k_3 \log (x) - k_1 \log (y)$ on orbits. In particular, the ODE system (2.6)–(2.7) admits periodic solutions, but it has no limit cycles. In Sect. 3, we will start our investigation of the existence and number of limit cycles of chemical reaction networks in sets ${{\mathbb {S}}}_n$ and ${{\mathbb {M}}}_n$. We will observe that ODE systems in ${{\mathbb {M}}}_2$ do not have any limit cycles, but there are reaction systems in ${{\mathbb {S}}}_2$ with limit cycles. There are also other important properties and classes of chemical reaction networks, including weakly reversible chemical systems (Craciun et al. 2020; Boros et al. 2020). To define weak reversibility, we embed the chemical reaction network (2.1) as a planar E-graph (i.e. Euclidean embedded graph, see Craciun (2019); Yu and Craciun (2018)) where the nodes have coordinates $(\alpha _i,\beta _i)$ and $(\gamma _i,\delta _i)$ and each reaction corresponds to the edge $(\alpha _i,\beta _i) \longrightarrow (\gamma _i,\delta _i)$. For example, the planar E-graph corresponding to the Lotka-Volterra chemical system (2.5) has three edges

$$\begin{aligned} (1,0) \rightarrow (2,0), \qquad (1,1) \rightarrow (0,2), \qquad (0,1) \rightarrow (0,0) \end{aligned}$$

and it is schematically shown in Fig. 1a. We say that a chemical reaction network is *weakly reversible* if every edge of the associated planar E-graph is a part of an oriented cycle. Clearly, Lotka-Volterra system is not weakly reversible.

**Example.** Consider the first-order chemical reaction network

2.13 $$\begin{aligned} \emptyset \; \mathop {\longrightarrow }^{1} \; X \,, \qquad \qquad X \; \mathop {\longrightarrow }^{1} \; Y \,, \qquad \qquad Y \; \mathop {\longrightarrow }^{1} \; \emptyset . \end{aligned}$$

Its associated planar E-graph is visualized in Fig. 1b, schematically showing three edges $(0,0) \rightarrow (1,0),$ $(1,0) \rightarrow (0,1)$ and $(0,1) \rightarrow (0,0)$ corresponding to the three reactions of chemical system (2.13). Since every edge of the associated planar E-graph is a part of an oriented cycle, chemical reaction network (2.13) is weakly reversible. The ODE system (2.3)–(2.4) corresponding to the chemical system (2.13) is a planar linear ODE system

2.14 $$\begin{aligned} \frac{\text{ d }x}{\text{ d }t} \,= &  \, 1 \, - \, x \,, \end{aligned}$$

2.15 $$\begin{aligned} \frac{\text{ d }y}{\text{ d }t} \,= &  \, x \, - \, y \,. \end{aligned}$$

Multiplying the right-hand side of the ODE system by polynomial $(1+x^2 y)$, we obtain the ODE system

$$\begin{aligned} \frac{\text{ d }x}{\text{ d }t} \,= &  \, (1 \, + \, x^2 y) (1 \, - \, x) \,,\\ \frac{\text{ d }y}{\text{ d }t} \,= &  \, (1 \, + \, x^2 y) (x \, - \, y) \,, \end{aligned}$$

which are the reaction rate equations for the chemical system consisting of reactions (2.13) and additional three reactions:

2.16 $$\begin{aligned} &  2 \, X \, + \, Y \; \mathop {\longrightarrow }^{1} \; 3 \, X \, + \, Y \,, \qquad 3 \, X \, + \, Y \, \; \mathop {\longrightarrow }^{1} \; 2 \, X \, + \, 2 \, Y \,, \nonumber \\ &  2 \, X \, + \, 2 \, Y \; \mathop {\longrightarrow }^{1} \; 2 \, X \, + \, Y . \end{aligned}$$

The associated planar E-graph is visualized in Fig. 1c. This example illustrates that the multiplication of the right-hand side of the reaction rate ODEs by a polynomial with positive coefficients results with multiple copies of the original associated E-graph. This will be further used in Sect. 4, where we study weakly reversible systems with algebraic limit cycles. Moreover, the chemical system consisting of six reactions (2.13) and (2.16) in Fig. 1c provides another example of a weakly reversible system.

### Definition 3

Let $n \in {{\mathbb {N}}}.$ We denote by ${{\mathbb {W}}}_{\!n}$ the subset of $\,{{\mathbb {M}}}_n$ which corresponds to chemical reaction networks (2.1) that are weakly reversible.

Considering an arbitrary chemical reaction network, it belongs to ${{\mathbb {S}}}_n$ where *n* is the order given by (2.2). Moreover, the corresponding ODEs satisfy the property that they can be obtained as reaction rate equations of a chemical system in ${{\mathbb {M}}}_{n+1}$. We formulate this result as our next lemma.

### Lemma 3

We have

- $$ {{\mathbb {W}}}_{\!n} \subset {\mathbb M}_n \subset {{\mathbb {S}}}_n \qquad \quad \text{ for } \text{ all } \quad n \in {{\mathbb {N}}} $$ ,
- $$ {{\mathbb {M}}}_2 \subset {{\mathbb {S}}}_2 \subset {{\mathbb {M}}_3} \subset {{\mathbb {S}}}_3 \subset {{\mathbb {M}}_4} \subset {{\mathbb {S}}}_4 \subset ... $$ .

### Proof

(a) This is a direct consequence of Definitions 1, 2 and 3 of ${{\mathbb {W}}}_{\!n},$ ${{\mathbb {M}}}_n$ and ${{\mathbb {S}}_n}.$

(b) We show that ${{\mathbb {S}}}_n \subset {\mathbb M}_{n+1}$, as follows. Recall that in the proof of Lemma 1 above we have been able to obtain the polynomial right-hand side of a chemical system in ${{\mathbb {S}}}_n$ by using only reactions of the form $iX + jY \rightarrow (i-1)X + jY$, $iX + jY \rightarrow (i+1)X + jY$, $iX + jY \rightarrow iX + (j-1)Y$, or $iX + jY \rightarrow iX + (j+1)Y$, with $i+j \le n$. Note now that for these types of reactions the largest stoichiometric coefficients are either $i+1$ and *j*, or *i* and $j+1$, and we have $i+1+j = i+j+1 \le \ n+1$. This implies that any system that belongs to ${{\mathbb {S}}}_n$ also belongs to ${{\mathbb {M}}}_{n+1}$. $\square$

## Hilbert number and its analogues for sets ${{\mathbb {S}}}_n$, ${{\mathbb {M}}}_n$ and ${{\mathbb {W}}}_{\!n}$

Let *H*(*n*) be the maximum number of limit cycles for planar ODE systems in the form (1.1)–(1.2), where *f* and *g* are polynomials of degree at most *n* given by (2.8). Then *H*(*n*) is often called the Hilbert number, because it can be used to formulate Hilbert’s 16th problem (Christopher and Li 2007). By constructing polynomial ODE systems with a specific number of limit cycles, lower bounds on the Hilbert number *H*(*n*) have been obtained: for example, a quadratic system with 4 limit cycles (Shi 1980), a cubic system with 13 limit cycles (Li et al. 2009) and a quartic system with 28 limit cycles (Prohens and Torregrosa 2018) have been constructed in the literature, giving $H(2) \ge 4$, $H(3) \ge 13$ and $H(4) \ge 28$. On the other hand, one can show that quadratic systems which can be written as chemical systems corresponding to bimolecular systems ${{\mathbb {M}}}_2$ cannot have limit cycles (Póta 1983; Schuman and Tóth 2003). To formulate our results and put them into context with the literature, we first define the corresponding counterparts of the Hilbert number *H*(*n*) for subsets of the polynomial ODE systems corresponding to sets ${{\mathbb {S}}}_n$, ${{\mathbb {M}}}_n$ and ${{\mathbb {W}}}_{\!n}$.

### Definition 4

We denote by *S*(*n*) the maximum number of limit cycles of ODEs in the set $\,{{\mathbb {S}}}_n$ of the *n*-th order chemical reaction networks, by *M*(*n*) the maximum number of limit cycles of ODEs in the set $\,{{\mathbb {M}}}_n$ of *n*-molecular chemical reaction networks, and by $W{\hspace{-0.85358pt}}(n)$ the maximum number of limit cycles of ODEs in the set ${{\mathbb {W}}}_{\!n}$ of weakly reversible chemical reaction networks.

**Table 1:** Some values and estimates from below on numbers $W{\hspace{-0.85358pt}}(n)$, *M*(*n*), *S*(*n*) and *H*(*n*), see Lemmas 5, 6, 7 and 8

*n*	$W{\hspace{-0.56905pt}}(n)$	*M*(*n*)	*S*(*n*)	*H*(*n*)
2	= 0	= 0	$\ge$ 3	$\ge$ 4
3	$\ge$ 3	$\ge$ 3	$\ge$ 6	$\ge$ 13
4	$\ge 6$	$\ge 6$	$\ge 13$	$\ge$ 28
as $n \rightarrow \infty$	$\ge {{\mathcal {O}}}(n^2 log(n))$	$\ge {{\mathcal {O}}}(n^2 log(n))$	$\ge {{\mathcal {O}}}(n^2 log(n))$	$\ge {{\mathcal {O}}}(n^2 log(n))$

Some inequalities between numbers $W{\hspace{-0.85358pt}}(n),$ *M*(*n*), *S*(*n*) and *H*(*n*) are stated as our next lemma.

### Lemma 4

We have

- $$W{\hspace{-0.85358pt}}(n) \le M(n) \le S(n) \le H(n)$$ for all $$n \in {{\mathbb {N}}}\,$$,
- $$ H(n) \le S(n+1)$$ for all $$n \in {{\mathbb {N}}}\,$$,
- $$S(n) \,\le M(n+1)$$ for all $$n \in {{\mathbb {N}}}\,$$,
- $$H(n) \le W(n+3)$$ for all $$n \in {{\mathbb {N}}}\,$$.

### Proof

(a) The first two inequalities follow directly from Lemma 3(a) and Definition 4 of numbers $W{\hspace{-0.85358pt}}(n),$ *M*(*n*) and *S*(*n*). The last inequality is a direct consequence of the definition of the Hilbert number *H*(*n*).

(b) First, it is easy to prove a weaker inequality that $H(n) \le S(n+2)$. For this, we observe that any *n*-degree polynomial ODE system (1.1)–(1.2) can be transformed into a chemical system of at most $(n+2)$-th order by shifting all limit cycles to the positive quadrant $[0,\infty )\times [0,\infty )$ and by multiplying both right-hand sides by *xy*. Since this corresponds to a rescaling of time in the original system, both the original system and the transformed system in ${{\mathbb {S}}}_{n+2}$ will have the same number of limit cycles. This implies that $H(n) \le S(n+2)$.

In order to prove that $H(n) \le S(n+1)$ we rely on the recent work of Plesa (2024), and also on the recent work of Gasull and Santana (2024). Consider first the case $H(n) < \infty$. Then, according to Theorem 2 in Gasull and Santana (2024), there exist ODE systems of degree *n* with exactly *H*(*n*) *hyperbolic* limit cycles. Then these limit cycles persist after small perturbations of the functions on the right-hand side of the ODEs; therefore, according to the results in Plesa (2024), we can transform these ODEs into chemical systems of at most $(n+1)$-th order which still have at least *H*(*n*) limit cycles. This implies the desired inequality for the case $H(n) < \infty$. If $H(n) = \infty$, then, again according to Theorem 2 in Gasull and Santana (2024), for any $k>0$ there exist ODE systems of degree *n* with at least *k* hyperbolic limit cycles, and, like before, this implies that $S(n+1) = \infty$.

(c) This inequality follows directly from Lemma 3(b). Note that these inequalities also trivially hold for $n=1$, because there are no limit cycles in linear systems, *i.e.,* $W(1)=M(1)=S(1)=H(1)=0$.

(d) Consider an ODE system of degree *n* that has *H*(*n*) limit cycles. Without loss of generality we can assume that the limit cycles lie in the positive quadrant, because we can shift this system via a linear change of variables, and the shift does not affect the degree. Let us now multiply the right-hand side of this system by *xy* as in the proof of part (b). The resulting system belongs to ${{\mathbb {S}}}_{n+2}$ and has the same *H*(*n*) limit cycles. According to results in Gasull and Santana (2024), there exists a small perturbation of this system that has *H*(*n*) *hyperbolic* limit cycles in the positive orthant, such that the monomials on the right-hand side of the perturbed system are the same as the monomials of the original unperturbed system. Therefore, the perturbed system still belongs to ${{\mathbb {S}}}_{n+2}$. Recall now that ${{\mathbb {S}}}_{n+2} \subset {{\mathbb {M}}}_{n+3}$. Now we do a second small perturbation of this system, in order to bring it from ${{\mathbb {M}}}_{n+3}$ to ${{\mathbb {W}}}_{n+3}$. In this perturbation we simply make each reaction reversible, but with a very small reaction rate constant for any new reaction that we add to the network. If these rate constants are small enough it follows that we obtain a system in ${{\mathbb {W}}}_{n+3}$ that has at least *H*(*n*) limit cycles, and therefore $H(n) \le W(n+3)$. $\square$

Some lower bounds on numbers $W{\hspace{-0.85358pt}}(n),$ *M*(*n*),  *S*(*n*) and *H*(*n*) can also be established. They are summarized in Table 1 and stated in lemmas below.

### Lemma 5

The ODEs in set ${{\mathbb {M}}}_2$ cannot have any limit cycles, i.e. we have $M(2) = 0$ and $W(2)=0.$

### Proof

See Póta (1983), Tyson and Light (1973), Schuman and Tóth (2003) for the proof of $M(2)=0$. Using Lemma 4, we have $W(2) \le M(2)$, which implies $W(2)=0.$ $\square$

### Lemma 6

We have $S(2) \ge 3,$ $S(3) \ge 6$, $S(4) \ge 13,$ $M(3) \ge 3$, $M(4) \ge 6$, $W(3) \ge 3$ and $W(4) \ge 6.$

### Proof

The fact that $S(2) \ge 1$ has been known for more than 80 years (Frank-Kamenetsky and Salnikov 1943), and is considered an important classical example in the mathematical theory of autocatalysis. The improved lower bound $S(2) \ge 3$ is due to Escher (1981). The results $S(3) \ge 6$ and $S(4) \ge 13$ have been established in the literature on Kolmogorov systems (Lloyd et al. 2002; Carvalho et al. 2023). A planar cubic (resp. quartic) ODE system with six (resp. thirteen) limit cycles in the positive quadrant has been presented in Lloyd et al. (2002) (resp. Carvalho et al. (2023)). Applying Lemma 1 to these systems, we conclude that there is a chemical system in ${{\mathbb {S}}}_3$ with six limit cycles and a chemical system in ${{\mathbb {S}}}_4$ with thirteen limit cycles, giving $S(3) \ge 6$ and $S(4) \ge 13$. Using Lemma 4(c), we get $M(3) \ge S(2) \ge 3$ and $M(4) \ge S(3) \ge 3.$ Finally, we can add reactions with very small values of rate constants to make the corresponding systems (weakly) reversible. Such small perturbations will not change the existence of hyperbolic limit cycles (Smale and Hirsch 1974; Perko 2013), giving $W(3) \ge 3$ and $W(4) \ge 6.$ $\square$

### Lemma 7

We have $H(2) \ge 4$, $H(3) \ge 13$, $H(4) \ge 28$ and $H(n) \ge {{\mathcal {O}}}(n^2 log(n))$ as $n \rightarrow \infty .$

### Proof

See Shi (1980) for $H(2) \ge 4$, Li et al. (2009) for $H(3) \ge 13$, Prohens and Torregrosa (2018) for $H(4) \ge 28$ and Christopher and Li (2007) for the asymptotic inequality $H(n) \ge {{\mathcal {O}}}(n^2 log(n))$ as $n \rightarrow \infty .$ $\square$

### Lemma 8

We have $S(n) \ge {{\mathcal {O}}}(n^2 log(n))$, $M(n) \ge {{\mathcal {O}}}(n^2 log(n))$ and $W(n) \ge {{\mathcal {O}}}(n^2 log(n))$, as $n \rightarrow \infty .$ .

### Proof

Using Lemma 4, we get $S(n) \ge H(n-1)$, $M(n) \ge S(n-1) \ge H(n-2)$ and $W(n) \ge H(n-3).$ The results then follow by applying the asymptotic inequality for *H*(*n*) given in Lemma 7. $\square$

The analysis of ODE systems with limit cycles which are used to achieve lower bounds in Table 1 often cannot be supported by illustrative numerical simulations, because some parameter values are negligible (beyond the machine precision) when compared to other parameter values. However, there are also chemical systems in the literature with multiple limit cycles, where the phase plane can be computed using standard numerical methods. For example, the third-order system in ${{\mathbb {S}}}_3$ with three limit cycles, two stable and one unstable, is presented in Plesa et al. (2017), and a 3-molecular chemical system in ${{\mathbb {M}}}_3$ with two limit cycles, one stable and one unstable is studied in Nagy et al. (2020).

In the following sections we will restrict our investigations to *algebraic* limit cycles, with a counterpart of Table 1 for algebraic limit cycles presented in Sect. 4. We also introduce a general approach in Theorem 3 in Sect. 5 to obtain chemical systems where we will be able to calculate their phase planes with multiple (algebraic) limit cycles and present some illustrative numerical results.

## Chemical systems with algebraic limit cycles

The analogues of the Hilbert number *H*(*n*) for polynomial ODE systems corresponding to the sets ${{\mathbb {S}}}_n$, ${{\mathbb {M}}}_n$ and ${{\mathbb {W}}}_{\!n}$ have been given in Definition 4. In this section, we will focus on algebraic limit cycles (Chavarriga et al. 2004; Gasull and Giacomini 2023). An algebraic limit cycle is not only a closed isolated solution of the ODE system, but it can also be represented as a closed component of an algebraic curve $h(x,y)=0$, where *h* is a polynomial of degree $n_h$. Note that, since the flow of the planar ODE system  (1.1)–(1.2) is tangent to the algebraic curve, we have

4.1 $$\begin{aligned} \frac{\partial h}{\partial x}(x,y) \, f(x,y) \, + \frac{\partial h}{\partial y}(x,y) \, g(x,y) \, = \, s(x,y) \, h(x,y) , \end{aligned}$$

where *s*(*x*, *y*) is a polynomial, called *cofactor* of *h*. First, we define versions of *S*(*n*),  *M*(*n*) and *W*(*n*) for counting only algebraic limit cycles.

### Definition 5

We denote by $S^a(n)$ the maximum number of algebraic limit cycles for ODEs in the set $\,{{\mathbb {S}}}_n$, by $M^a(n)$ the maximum number of algebraic limit cycles for ODEs in the set $\,{{\mathbb {M}}}_n$ of *n*-molecular chemical reaction networks, and by $W^a(n)$ the maximum number of algebraic limit cycles for ODEs in the set ${{\mathbb {W}}}_{\!n}$ of weakly reversible chemical reaction networks.

A counterpart of Lemma 4 establishing inequalities between numbers $W{\hspace{-0.85358pt}}(n),$ *M*(*n*), *S*(*n*) and *H*(*n*) can also be formulated for numbers $W^a(n),$ $M^a(n)$, $S^a(n)$ and $H^a(n)$ counting only algebraic limit cycles.

### Lemma 9

We have

- $$W^a(n) \le M^a(n) \le S^a(n) \le H^a(n) \le S^a(n+2)$$ for all $$n \in {{\mathbb {N}}}\,$$,
- $$S^{(a)}(n) \le M^{(a)}(n+1)$$ for all $$n \in {{\mathbb {N}}}\,$$.

### Proof

The proof follows some of the same arguments as in the proof of Lemma 4, where we replace limit cycles by algebraic limit cycles. $\square$

Some lower bounds on numbers $W^a(n)$, $M^a(n)$ and $S^a(n)$ are given in Table 2 and in Lemmas 10, 11 and 12.

**Table 2:** Some values and estimates from below on numbers $W^a(n)$, $M^a(n)$, $S^a(n)$ and $H^a(n)$, see Lemma 10, Lemma 11, Lemma 12 and Theorem 1

*n*	$W^a{\hspace{-0.56905pt}}(n)$	$M^a(n)$	$S^a(n)$	$H^a(n)$
2	= 0	= 0	$\ge$ 1	$\ge$ 1
3	$\ge$ 0	$\ge$ 1	$\ge$ 1	$\ge$ 2
4	$\ge$ 1	$\ge$ 1	$\ge$ 3	$\ge$ 4
as $n \rightarrow \infty$	$\ge {{\mathcal {O}}}(n)$	$\ge {{\mathcal {O}}}(n^2$)	$\ge {{\mathcal {O}}}(n^2$)	$\ge {{\mathcal {O}}}(n^2$)

### Lemma 10

We have $H^a(2) \ge 1$, $H^{(a)}(3) \ge 2$, $H^a(4) \ge 4,$ $S^a(2) \ge 1$, $S^{(a)}(3) \ge 1$, $S^a(4) \ge 3,$ $M^{(a)}(2) = 0,$ $M^a(3) \ge 1,$ $M^{(a)}(4) \ge 1$ and $W^{(a)}(2) = 0.$

### Proof

See Llibre et al. (2010) for $H^a(2) \ge 1$, $H^{(a)}(3) \ge 2$ and $H^a(4) \ge 4.$ Since Lemma 5 gives $M(2) = 0$ and $W(2)=0$, we also have $M^{(a)}(2) = 0$ and $W^{(a)}(2) = 0$ when we restrict to algebraic limit cycles in sets ${{\mathbb {M}}}_2$ and ${{\mathbb {W}}}_2$. To show $S^a(2) \ge 1$, we need to find a quadratic chemical system with an algebraic limit cycle. Consider the quadratic system (Escher 1979)

4.2 $$\begin{aligned} \frac{\text{ d }x}{\text{ d }t}= &  2x^2 - xy + \frac{3}{2}, \end{aligned}$$

4.3 $$\begin{aligned} \frac{\text{ d }y}{\text{ d }t}= &  \frac{5}{2}x^2 - xy - y + \frac{17}{4}. \end{aligned}$$

Using Lemma 1, the ODE system (4.2)–(4.3) belongs to ${{\mathbb {S}}}_2.$ Moreover, it is easy to verify that it has an algebraic limit cycle in the positive quadrant, which is the ellipse given by Escher (1979)

4.4 $$\begin{aligned} h(x,y) = 10x^2 - 12xy + 4y^2 + 20x - 16y + 19 = 0 \end{aligned}$$

with the cofactor *s*(*x*, *y*) in equation (4.1) given as $s(x,y)=x-2.$ Consequently, we have $S^a(2) \ge 1$. Using Lemma 9, we conclude

4.5 $$\begin{aligned} 1 \le S^{(a)}(2) \le M^{(a)}(3) \le S^{(a)}(3) \le M^{(a)}(4). \end{aligned}$$

Finally, to show $S^a(4) \ge 3,$ consider quartic algebraic curve $h(x,y)=0$ given by

**Fig. 2:**
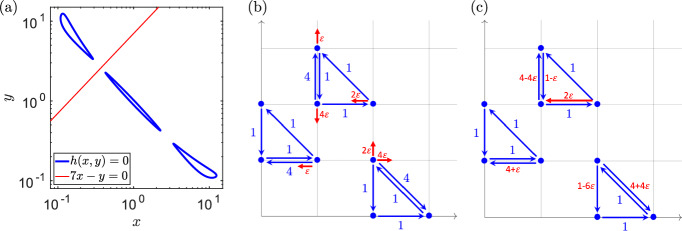
(**a**) The quartic algebraic curve $h(x,y)=0$ given by (4.6) has three (blue) ovals in the positive quadrant. The red line is given by $7x-y=0$. The red line does not intersect the (blue) ovals of $h(x,y)=0$ in the positive quadrant. We use log scale on the *x*-axis and *y*-axis. (**b**) The chemical reaction network corresponding to the blue edges of the planar E-graph realizes the ‘unperturbed’ ODE system (4.11)–(4.12), while the $\varepsilon$-perturbations are shown by the red edges. (**c**) The weakly reversible network that consists of the blue edges (with some modified rate constants) together with the red edge provides a weakly reversible realization of the perturbed system (4.16)–(4.17) (color figure online)

4.6 $$\begin{aligned} h(x,y) \,= &  \, x^{2}y^{2} \, - \, \frac{9}{10^3} \, \big ( x^{3}y + xy^{3} \big ) \, + \, \frac{6}{10^4} \, \big ( x^{3}+y^{3} \big ) \, \nonumber \\ &  + \, \frac{2}{50} \, \big ( x^{2} y + x y^{2} \big ) \, - \, 2 xy \, + \, \frac{934}{10^3} \,, \end{aligned}$$

which has three ovals in the positive quadrant. We visualize them in Fig. 2a. Next, we consider the line $\, y = 7 x \,$, which does not intersect the three ovals of $h(x,y)=0$, as it is shown in Fig. 2a. Then, we can apply (Christopher 2001, Theorem 1) to deduce that the ODE system

4.7 $$\begin{aligned} \frac{{\textrm{d}}x}{{\textrm{d}}t} \,= &  \, h(x,y) \, + \, (y - 7 x) \, \frac{\partial h}{\partial y}(x,y) , \end{aligned}$$

4.8 $$\begin{aligned} \frac{{\textrm{d}}y}{{\textrm{d}}t} \,= &  \, h(x,y) \, + \, (7 x - y ) \, \frac{\partial h}{\partial x}(x,y) , \end{aligned}$$

is a polynomial system of degree 4 which has the three ovals of $h(x,y)=0$ in the positive quadrant as hyperbolic algebraic limit cycles. Using Lemma 1, we observe that the ODE system (4.7)–(4.8) is in set ${{\mathbb {S}}}_4$. Therefore, we conclude that $S^a(4) \ge 3.$ $\square$

In Lemma 10, we have presented a second-order chemical system in ${{\mathbb {S}}}_2$ with the algebraic limit cycle given as ellipse (4.4). The corresponding ODE system (4.2)–(4.3) has quadratic polynomials on the right-hand side. Another quadratic polynomial dynamical system with an algebraic limit cycle is (Chavarriga et al. 2004)

4.9 $$\begin{aligned} \frac{\text{ d }x}{\text{ d }t} \,= &  \, 2 \, \big (1 \, + \,2\,x \, - \, 2 \, c \, x^2 \, + \, 6 \,x\,y \big ), \end{aligned}$$

4.10 $$\begin{aligned} \frac{\text{ d }y}{\text{ d }t} \,= &  \, 8 \, - \, 3 \, c \, - \, 14\,c\,x \, - \, 2 \, c \, x\,y \, - \, 8 \, y^2 \,. \end{aligned}$$

As explained in Chavarriga et al. (2004), for any $c \in (0,1/4)$ the ODE system (4.9)–(4.10) has an invariant algebraic curve, which is a limit cycle (Gasull and Giacomini 2023). The ODE system (4.9)–(4.10) is not a chemical system because of the term $-14 {\hspace{0.56905pt}} c {\hspace{0.56905pt}} x$ in equation (4.10). However, we can use this quadratic system for $c = 1/8$ to construct a cubic mass-action system that has an algebraic limit cycle. For this we note that the limit cycle of the quadratic system (4.9)–(4.10) is contained in the set $(0, \infty ) \times (-1, \infty )$, and therefore if we shift this system one unit in the *y*-direction, and then multiply its right-hand side by a factor of *y*, we obtain

$$\begin{aligned} \frac{\text{ d }x}{\text{ d }t}= &  2 \, \big (1 \, + \,2\,x \, - \, 2 \, c \, x^2 \, + \, 6 \,x \, (y-1) \big ) \, y, \\ \frac{\text{ d }y}{\text{ d }t}= &  \, \big ( 8 \, - \, 3 \, c \, - \, 14\,c\,x \, - \, 2 \, c \, x \, (y-1) \, - \, 8 \, (y-1)^2 \big ) \, y \,. \end{aligned}$$

Then, the cubic system above is a mass-action system; moreover, it has an algebraic limit cycle, because its trajectory curves are the shifted versions of the trajectory curves of the system (4.9)–(4.10). Such a construction provides an alternative way for us to show that $S^{(a)}(3) \ge 1$, which we have previously established in the proof of Lemma 10 by using inequalities (4.5).

In Sect. 3, it has been relatively straightforward to establish that weakly reversible chemical systems can give rise to limit cycles by using small perturbations of non-reversible chemical systems. This is not the case, when we consider algebraic limit cycles, because a small perturbation can change an algebraic limit cycle to a non-algebraic one. In our next lemma, we show that weakly reversible chemical systems can have algebraic limit cycles by constructing a quartic weakly reversible two-species system. The question of whether cubic weakly reversible two-species systems can give rise to algebraic limit cycles remains open, leaving us with inequality $W^{(a)}(3) \ge 0$ in Table 2. In our construction we rely on a general approach for constructing weakly reversible systems that have a curve of equilibria; this approach has been introduced in Boros et al. (2020).

### Lemma 11

We have $$W^a(4) \ge 1.$$

### Proof

We will construct a weakly reversible system that has an algebraic limit cycle, as follows. We start with a weakly reversible chemical system constructed in reference Boros et al., (2020, Example 4.3), given by

4.11 $$\begin{aligned} \frac{\text{ d }x}{\text{ d }t}= &  \big (x^2+xy^2+y-4xy\big ) \, (1-x), \end{aligned}$$

4.12 $$\begin{aligned} \frac{\text{ d }y}{\text{ d }t}= &  \big (x^2+xy^2+y-4xy\big )\,(x-y). \end{aligned}$$

The common factor

4.13 $$\begin{aligned} h(x,y) = x^2+xy^2+y-4xy \end{aligned}$$

results in a curve of positive steady states within the positive quadrant. Note that the polynomials on the right-hand side of the ODE system (4.11)–(4.12) have degree 4. They can be obtained by multiplying the linear system (2.14)–(2.15) by (4.13). In Fig. 1c, we illustrated that the multiplication of the linear system (2.14)–(2.15) by positive monomials results in copies of the network. Generalizing this observation to the ODE system (4.11)–(4.12), we can realize it by the chemical reaction network shown in blue in Fig. 2b or Fig. 2c. We now consider a perturbed version of the ODE system (4.11)–(4.12), also of degree 4, given by

4.14 $$\begin{aligned} \frac{\text{ d }x}{\text{ d }t}= &  \big (x^2+xy^2+y-4xy\big )\,(1-x) \, - \, \varepsilon \, x \, y \, \frac{\partial h}{\partial y}(x,y) , \end{aligned}$$

4.15 $$\begin{aligned} \frac{\text{ d }y}{\text{ d }t}= &  \big (x^2+xy^2+y-4xy\big )\,(x-y) \, + \, \varepsilon \, x \, y \, \frac{\partial h}{\partial x}(x,y) , \end{aligned}$$

which implies

4.16 $$\begin{aligned} \frac{\text{ d }x}{\text{ d }t}= &  \big (x^2+xy^2+y-4xy\big )\,(1-x) \, - \, \varepsilon \, x \, y \, \big (2xy +1 - 4x\big ), \end{aligned}$$

4.17 $$\begin{aligned} \frac{\text{ d }y}{\text{ d }t}= &  \big (x^2+xy^2+y-4xy\big )\,(x-y) \, + \, \varepsilon \, x \,y \, \big (2x + y^2 -4y\big ). \end{aligned}$$

The ODE system (4.16)–(4.17) has an algebraic limit cycle given by $h(x,y)=0$. One possible way to check that the periodic solution that lies along the curve $h(x,y)=0$ is indeed a limit cycle is to look at a more general setting which is discussed in depth in Sect. 5; specifically, it is easy to check that the transversality condition (5.6) holds for the ODE system (4.16)–(4.17).

The red edges in Fig. 2b suggest a realization of the ODE system (4.16)–(4.17) as a chemical reaction network in ${{\mathbb {S}}}_4$ for any $\varepsilon > 0$, but this particular realization is not weakly reversible. However, reaction network realizations of polynomial dynamical systems are not unique in general (Craciun and Pantea 2008; Plesa et al. 2018; Craciun et al. 2020). If $\varepsilon \in (0, 1/6)$, then there do exist weakly reversible realizations of the ODE system (4.16)–(4.17); one such realization is shown in Fig. 2c. Therefore, the ODE system (4.16)–(4.17) is in ${{\mathbb {W}}}_4$ for $\varepsilon \in (0, 1/6)$, giving $W^a(4) \ge 1.$ $\square$

### Lemma 12

We have $H^a(n) \ge {{\mathcal {O}}}(n^2)$, $S^a(n) \ge {{\mathcal {O}}}(n^2)$ and $M^a(n) \ge {{\mathcal {O}}}(n^2)$, as $n \rightarrow \infty .$

### Proof

See Llibre et al. (2010) for $H^a(n) \ge {{\mathcal {O}}}(n^2)$ as $n \rightarrow \infty .$ The next two asymptotic inequalities follow from Lemma 9. $\square$

Lemmas 10, 11 and 12 have justified all lower bounds in Table 2, except of the asymptotic inequality $W^a(n) \ge {{\mathcal {O}}}(n)$ as $n \rightarrow \infty .$ We will show this inequality in the next subsection by considering reversible chemical systems, which is even more restrictive class of chemical reaction networks than weakly reversible systems. In particular, we will show that reversible chemical systems can have (multiple) algebraic limit cycles.

### Algebraic limit cycles for reversible chemical systems

**Fig. 3:**
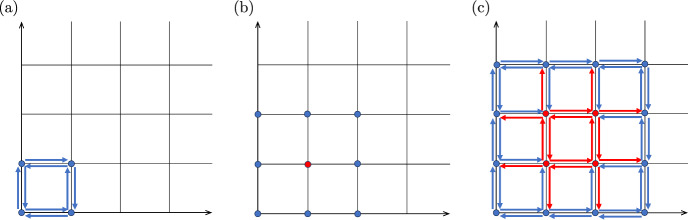
(**a**) Planar E-graph of a reversible chemical reaction network corresponding to the ODE system (4.18)–(4.19) as its reaction rate equations with all reaction rate constants equal to 1. (**b**) A geometric representation of the monomials of the polynomial *h*(*x*, *y*) given by (4.20). The blue points represent the monomials with positive coefficients and the red point represents its negative monomial $-9xy$. **c** The dynamical system obtained by multiplying the network shown in **a** by the factor *h*(*x*, *y*) shown in (**b**) (which gives rise to the equations (4.21)–$(4.22))$ can be realized by this reversible ‘full-grid’ network (color figure online)

In this section we describe a general approach for constructing reversible systems with algebraic limit cycles. We start with the simple chemical reaction network shown in Fig. 3a. If we choose all the reaction rate constants to be equal to 1, the corresponding reaction rate equations are given by the ODE system

4.18 $$\begin{aligned} \frac{\text{ d }x}{\text{ d }t}&= 1-x+y-xy, \end{aligned}$$

4.19 $$\begin{aligned} \frac{\text{ d }y}{\text{ d }t}&= 1+x-y-xy, \end{aligned}$$

which has a globally attracting point at $(x,y)=(1,1)$. Next, we consider the algebraic curve $h(x,y)=0$ of degree $n_h=4$ given by

**Fig. 4:**
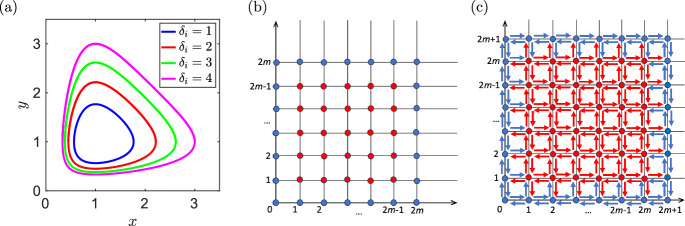
(**a**) The algebraic curve $h(x,y)=0$ given by (4.20) is plotted as the blue line, together with algebraic curves $h_i(x,y)=0$ given by (4.27) for $\delta _i = 2$ (red line), $\delta _i=3$ (green line) and $\delta _i = 4$ (magenta line). (**b**) The negative coefficients of product $h_0$ given by (4.28) correspond to (a subset of) monomials that are represented by the red points, while the coefficients of the monomials that are represented by the blue points are all positive. (**c**) A reversible chemical reaction network which can be modelled by the reaction rate equations written in the form of the ODE system (4.29)–(4.30), which has *N* algebraic limit cycles (color figure online)

4.20 $$\begin{aligned} h(x,y) = x^2y^2+x^2y+xy^2+x^2+y^2+x+y+1-9xy\,, \end{aligned}$$

then, within the positive quadrant, the equation $h(x,y)=0$ is satisfied along a simple closed curve. Indeed, we can rewrite $h(x,y)=0$ as

$$\begin{aligned} \left( x + 1 + \frac{1}{x} \right) \left( y + 1 + \frac{1}{y} \right) = 10, \end{aligned}$$

which has two solutions *y* for each *x* satisfying $(7-\sqrt{13})/6< x < (7+\sqrt{13})/6$. Plotting the values of *y* as functions of *x* in Fig. 4a, we obtain the two branches of the closed curve visualized as the blue line. A geometric representation of the monomials of *h*(*x*, *y*) in (4.20) is shown in Fig. 3b. Multiplying the right-hand side of the ODE system (4.18)–(4.19) by *h*(*x*, *y*), we obtain the ODE system

4.21 $$\begin{aligned} \frac{\text{ d }x}{\text{ d }t}= &  \big (x^2y^2+x^2y+xy^2+x^2+y^2+x+y+1-9xy\big ) \big (1-x+y-xy\big ), \nonumber \\ \end{aligned}$$

4.22 $$\begin{aligned} \frac{\text{ d }y}{\text{ d }t}= &  \big (x^2y^2+x^2y+xy^2+x^2+y^2+x+y+1-9xy\big ) \big (1+x-y-xy\big ), \nonumber \\ \end{aligned}$$

which has polynomials of degree 6 on the right hand side. The ODE system (4.21)–(4.22) has a curve of equilibria (Boros et al. 2020) given by $h(x,y)=0$. Moreover, since the ODE system (4.21)–(4.22) has been obtained by multiplying the ODE system (4.18)–(4.19) by a polynomial, the corresponding planar E-graph representation will consists of shifted copies of the reaction network in Fig. 3a for each multiplication by a positive monomial, as we have already observed in Fig. 1c.

We argue that the ODE system (4.21)–(4.22) has a weakly reversible realization given by the network in Fig. 3c, and, moreover, this realization can be chosen such that all reactions have reaction rate constants $\ge 1$. For this, we first note that the reactions shown in *blue* in Fig. 3c can all be chosen to have reaction rate constants equal to 1, because these are obtained from reactions in Fig. 3a after multiplying with one of the *positive* monomials of *h*(*x*, *y*).

On the other hand, the reactions shown in *red* in Fig. 3c may have rate constants that are impacted by multiplication with some positive and some negative monomials of *h*(*x*, *y*), so their size (and even their sign) are not immediately clear. Nevertheless, note that, no matter what values these rate constants have to begin with, if we increase all of them by an arbitrarily chosen constant, then *the effect of all these increases cancels out.* This is due to the fact that the red reactions can be partitioned into pairs, such that each pair of reactions originates at the same red node, and the two reactions within each such pair point exactly opposite from each other. Therefore, we conclude that the system (4.21)–(4.22) can be realized by the network shown in Fig. 3c. Consider now a perturbed version of this system, also of degree 6, given by

4.23 $$\begin{aligned} \frac{\text{ d }x}{\text{ d }t}= &  \big (x^2y^2+x^2y+xy^2+x^2+y^2+x+y+1-9xy\big )\,\big (1-x+y-xy\big ) \,\nonumber \\ &  - \, \varepsilon \, x \, y \, \frac{\partial h}{\partial y}(x,y) , \end{aligned}$$

4.24 $$\begin{aligned} \frac{\text{ d }y}{\text{ d }t}= &  \big (x^2y^2+x^2y+xy^2+x^2+y^2+x+y+1-9xy\big )\, \big (1+x-y-xy\big ) \,\nonumber \\ &  + \, \varepsilon \, x \, y \, \frac{\partial h}{\partial x}(x,y) , \end{aligned}$$

which implies

4.25 $$\begin{aligned} \frac{\text{ d }x}{\text{ d }t}= &  \big (x^2y^2+x^2y+xy^2+x^2+y^2+x+y+1-9xy\big )\, \big (1-x+y-xy\big ) \nonumber \\ &  - \, \varepsilon \, x \, y \big (2x^2y + x^2+2xy+2y+1-9x\big ) , \end{aligned}$$

4.26 $$\begin{aligned} \frac{\text{ d }y}{\text{ d }t}= &  \big (x^2y^2+x^2y+xy^2+x^2+y^2+x+y+1-9xy\big )\,\big (1+x-y-xy\big ) \nonumber \\ &  + \, \varepsilon \, x \, y \, \big (2xy^2+ y^2+2xy+2x+1-9y\big ) \,. \end{aligned}$$

The ODE system (4.25)–(4.26) has been constructed in a similar way as the ODE system (4.16)–(4.17). Like in that example, it is easy to check that the transversality condition (5.6) also holds in this case. We therefore conclude that the ODE system (4.25)–(4.26) has an algebraic limit cycle, plotted as the blue line in Fig. 4a.

We now explain why this system also has a weakly reversible realization given by the network in Fig. 3c. Recall that we have observed above that the ODE system (4.21)–(4.22) has a realization that uses the reaction network in Fig. 3c with all reactions having reaction rate constants $\ge 1$. Note now that all the monomials in (4.25)–(4.26) that contain a factor of $\varepsilon$ already appear among the monomials of the ODE system (4.21)–(4.22), and if we choose $\varepsilon$ sufficiently small, the ODE system (4.25)–(4.26) can then be realized by the same reaction network as the ODE system (4.21)–(4.22). Therefore, the ODE system (4.25)–(4.26) can be realized by the reversible network shown in Fig. 3c.

Our construction of a reversible chemical system (4.25)–(4.26) with an algebraic limit cycle can be generalized to obtain reversible systems with multiple limit cycles. To do that, we replace a single factor *h*(*x*, *y*) by a product of several such factors. We construct reversible systems with several algebraic limit cycles in our next theorem.

#### Theorem 1

There exists a reversible chemical system of order $4N+2$ that has *N* algebraic limit cycles for all $N \in {{\mathbb {N}}}.$ In particular, we have $W^a(4N+2) \ge N$.

#### Proof

We define

4.27 $$\begin{aligned} h_i(x,y)= &  x^2y^2+x^2y+xy^2+x^2+y^2+x+y+1-(8+\delta _i) \, x \, y \,, \nonumber \\ &  \text{ for } \quad i=1,2,\dots , N , \end{aligned}$$

and for some mutually distinct real positive numbers $\delta _1,$ $\delta _2,$ $\dots ,$ $\delta _N$. Then the equation $h_i(x,y) = 0$ can be rewritten as

$$\begin{aligned} \left( x + 1 + \frac{1}{x} \right) \left( y + 1 + \frac{1}{y} \right) = \delta _i + 9 , \end{aligned}$$

which has two solutions for

$$\begin{aligned} 1 \, + \, \frac{\delta _i \,- \,\sqrt{\delta _i (12 + \delta _i)}}{6} \,<\, x \,<\, 1 \, + \, \frac{\delta _i \, + \,\sqrt{\delta _i (12 + \delta _i)}}{6} \end{aligned}$$

giving a simple closed curve in the positive quadrant for each $\delta _i>0$. As an illustration, we visualize four such curves in Fig. 4a for $\delta _i=1$, $\delta _i=2$, $\delta _i=3$ and $\delta _i=4.$ We note that $h_i$ given by (4.27) is equal to *h* given by (4.20) for $\delta _i=1$, which is plotted as the blue line in Fig. 4a. In particular, we note that $h_i(x,y)=0$ in equation (4.27) for mutually distinct real positive numbers $\delta _1,$ $\delta _2,$ $\dots ,$ $\delta _N$ give rise to *N* disjoint algebraic curves in the positive quadrant. Now denote

4.28 $$\begin{aligned} h_0 = \prod _{i=1}^N h_i \end{aligned}$$

and consider the system

4.29 $$\begin{aligned} \frac{\text{ d }x}{\text{ d }t}= &  h_0(x,y) \, \big (1-x+y-xy\big ) \, - \, \varepsilon \, x \, y \, \frac{\partial h_0}{\partial y}, \end{aligned}$$

4.30 $$\begin{aligned} \frac{\text{ d }y}{\text{ d }t}= &  h_0(x,y) \, \big (1+x-y-xy\big ) \, + \, \varepsilon \, x \, y \, \frac{\partial h_0}{\partial x}. \end{aligned}$$

The curves of the form $h_i(x,y) = 0$ lie along periodic trajectories of the system (4.29)–(4.30), and a quick way to ensure that each one of the curves $h_i(x,y) = 0$ is actually a limit cycle is to check the transversality condition (5.6) in Theorem 2. This can be done without additional calculations (by relying on the case of the ODE system (4.25)–(4.26)) if we assume that we have chosen all the $\delta _i$ to be close enough to 1.

The polynomial $h_0$ has degree 4*N*. From the definition of $h_0$ we conclude that if a monomial $x^p y^q$ of $h_0$ has a *negative* coefficient, then we have $1 \le p \le 4N-1$ and $1 \le q \le 4N-1$. This situation is illustrated in Fig. 4b, as follows: if the points in Fig. 4b represent monomials of $h_0$, then all the negative monomials are among the red points, and all the blue points correspond to *positive* monomials. Using the same argument as in Fig. 3b and c, we conclude that the ODE system (4.29)–(4.30) has a reversible realization which uses the reaction network illustrated in Fig. 4c, and the ODE system (4.29)–(4.30) has at least *N* algebraic limit cycles in the positive quadrant, given by the equations $h_i(x,y) = 0$. Therefore we have $W^a(4N+2) \ge N$. $\square$

## Robust limit cycles

The reaction rate equations (4.14)–(4.15), (4.23)–(4.24) and (4.29)–(4.30) can be written in the following general form

5.1 $$\begin{aligned} \frac{\text{ d }x}{\text{ d }t}= &  h(x,y) \, f_0(x,y) \, - \, \varepsilon \, x \, y \, \frac{\partial h}{\partial y}(x,y) , \end{aligned}$$

5.2 $$\begin{aligned} \frac{\text{ d }y}{\text{ d }t}= &  h(x,y) \, g_0(x,y) \, + \, \varepsilon \, x \, y \, \frac{\partial h}{\partial x}(x,y) , \end{aligned}$$

where $f_0(x,y),$ $g_0(x,y)$ and *h*(*x*, *y*) are polynomials. For example, the ODE system (4.14)–(4.15) is given in the general form (5.1)–(5.2) for

5.3 $$\begin{aligned} h(x,y) = x^2 + x y^2 + y - 4xy, \quad f_0(x,y) = 1-x \quad \text{ and } \quad g_0(x,y) = x-y.\nonumber \\ \end{aligned}$$

**Fig. 5:**
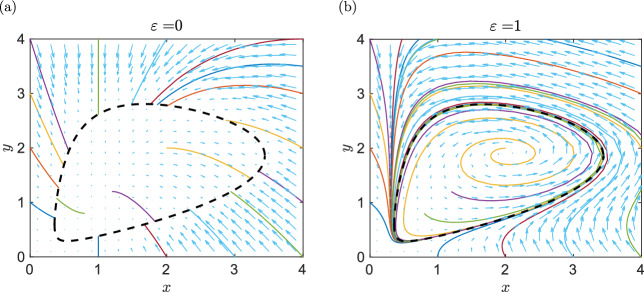
(**a**) The phase plane of the ODE system (4.11)–(4.12), i.e. the ODE system (4.16)–(4.17) for $\varepsilon =0$. We plot the algebraic curve $h(x,y)\equiv x^2 + x y^2 + y - 4xy = 0$ (black dashed line) together with some illustrative trajectories starting at the boundary of the visualized square. All trajectories converge to a stable steady state on the curve $h(x,y)=0.$ (**b**) The phase plane of the ODE system (5.4)–(5.5), i.e. the ODE system (4.16)–(4.17) for $\varepsilon =1$. The algebraic curve $h(x,y)=0$ becomes a stable limit cycle for $\varepsilon >0$

Substituting $\varepsilon =0$, we get the ODE system (4.11)–(4.12), which can be realized as a chemical system and has a continuum of stable steady states, given by $h(x,y)=0$. We illustrate this in Fig. 5a, where we plot the set $h(x,y)=0$ as the black dashed line together with fifteen illustrative trajectories starting at the boundary of the visualized domain $[0,4] \times [0,4]$ and three illustrative trajectories starting inside the oval $h(x,y)=0$. We observe that all calculated trajectories approach an equilibrium point inside the set $h(x,y)=0$ as $t \rightarrow \infty .$

In the proof of Lemma 11, we have found a weakly reversible realization of the ODE system (4.16)–(4.17) for $\varepsilon \in (0, 1/6)$. However, the ODE system (4.16)–(4.17) can be realized as a chemical system for all $\varepsilon \ge 0.$ For example, if $\varepsilon =1$, it simplifies to

5.4 $$\begin{aligned} \frac{\text{ d }x}{\text{ d }t}= &  x^2 + x y^2 + y - 6xy + 8x^2y - 3 x^2y^2 -x^3, \end{aligned}$$

5.5 $$\begin{aligned} \frac{\text{ d }y}{\text{ d }t}= &  x^3 + x^2 y^2 + x y - 3x^2y - y^2. \end{aligned}$$

This ODE system has one stable limit cycle as illustrated in Fig. 5b, where we calculate trajectories for the same initial conditions as in Fig. 5a. We observe that all calculated trajectories approach the stable limit cycle $h(x,y)=0.$ The existence of a stable limit cycle can also be established for the general system (5.1)–(5.2). We formulate it as our next theorem.

### Theorem 2

Consider the ODE system (5.1)–(5.2), where $f_0(x,y),$ $g_0(x,y)$ and *h*(*x*, *y*) are real polynomials. Assume that the set where $h(x,y) = 0$ contains *N* isolated simple closed curves, and also assume that the transversality condition

5.6 $$\begin{aligned} f_0(x,y) \, \frac{\partial h}{\partial x}(x,y) \, + \, g_0(x,y) \, \frac{\partial h}{\partial y}(x,y) \, \ne \, 0 \end{aligned}$$

holds along all these *N* isolated closed curves.

Then any simple closed curve where $h(x,y) = 0$ and

5.7 $$\begin{aligned} f_0(x,y) \, \frac{\partial h}{\partial x}(x,y) \, + \, g_0(x,y) \, \frac{\partial h}{\partial y}(x,y) \, < \, 0 \end{aligned}$$

is a stable algebraic limit cycle of the ODE system (5.1)–(5.2), for all values of $\varepsilon > 0$. Similarly, any simple closed curve where $h(x,y) = 0$ and

5.8 $$\begin{aligned} f_0(x,y) \, \frac{\partial h}{\partial x}(x,y) \, + \, g_0(x,y) \, \frac{\partial h}{\partial y}(x,y) \, > \, 0 \end{aligned}$$

is an unstable algebraic limit cycle of the ODE system (5.1)–(5.2), for all values of $\varepsilon > 0$.

In particular, the ODE system (5.1)–(5.2) has *N* algebraic limit cycles, for all values of $\varepsilon > 0$.

### Proof

The transversality condition (5.6) implies that the gradient of *h* does not vanish along the curve $h(x,y) = 0$. In particular, any simple closed curve where $h(x,y) = 0$ is a smooth curve. Note that the vector field

$$\begin{aligned} (-h_y,h_x) \equiv \left( - \frac{\partial h}{\partial y}, \frac{\partial h}{\partial x} \right) \end{aligned}$$

always points *along* any curve of the form $h(x,y) = \alpha$, i.e. never points *across* it; the same is true of the vector field $\varepsilon {\hspace{0.56905pt}} x {\hspace{0.56905pt}} y {\hspace{0.56905pt}} (-h_y, h_x)$. Therefore, the dynamics of the system (5.1)–(5.2) across curves of the form $h(x,y) = \alpha$ is determined by the vector field $(f_0, g_0)$.

Let us focus on the case where the condition (5.7) is satisfied along one such curve ${\mathcal {C}}$. (The other case is completely analogous.) Then there exists an annular neighborhood of ${\mathcal {C}}$ denoted ${\mathcal {A}}_{{\mathcal {C}}}(\delta )$, which is delimited by two curves where $h(x,y) = \pm \delta$, for some small number $\delta >0$, such that ${\mathcal {A}}_{{\mathcal {C}}}(\delta )$ is forward invariant for the system (5.1)–(5.2). To show this, we observe that the condition (5.7) implies that, for $\delta$ small enough, the two boundary curves of ${\mathcal {A}}_{{\mathcal {C}}}(\delta )$ where $h(x,y) = \pm \delta$ are smooth and (5.7) holds along them. Therefore, along the two boundary curves of ${\mathcal {A}}_{{\mathcal {C}}}(\delta )$ the vector field (5.1)–(5.2) points towards the interior of ${\mathcal {A}}_{{\mathcal {C}}}(\delta )$.

Moreover, if we fix some $\delta _0 >0$ such that $A_{{\mathcal {C}}}(\delta )$ is forward invariant for the system (5.1)–(5.2) for all $\delta \in (0,\delta _0]$, then it follows that ${\mathcal {A}}_{{\mathcal {C}}}(\delta _0)$ cannot contain any periodic orbit other than ${\mathcal {C}}$, and cannot contain any fixed point. Therefore, all the forward trajectories that start within ${\mathcal {A}}_{{\mathcal {C}}}(\delta _0)$ must converge to ${\mathcal {C}}$, which implies that ${\mathcal {C}}$ is a stable limit cycle of the system (5.1)–(5.2). $\square$

The vector field $(f_0,g_0)$ given by (5.3) has a single critical point (1, 1) inside the oval $h(x,y)=0$, and the transversality condition (5.6) is satisfied in our example in Fig. 5b. Such an approach is also used in our proof of Theorem 1 to obtain the ODE system (4.29)–(4.30), where we have

5.9 $$\begin{aligned} f_0(x,y) \, = \, 1-x+y-xy \, \qquad \text{ and } \qquad g_0(x,y) \, = \, 1+x-y-xy. \end{aligned}$$

**Fig. 6:**
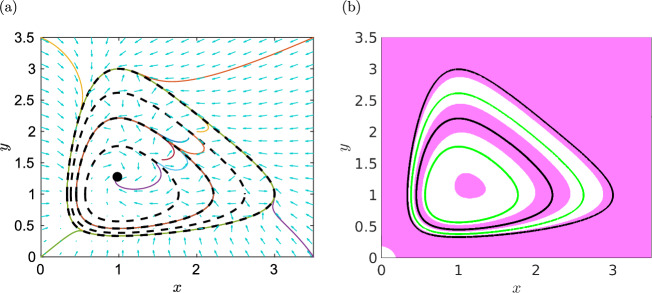
(**a**) The phase plane of the ODE system (4.29)–(4.30) for $\varepsilon =0.1$. We plot the algebraic curve $h_0(x,y)$ (black dashed line) together with ten illustrative trajectories. (**b**) The visualization of domains $\Omega _s$ (magenta shading) and $\Omega _u$ (white shading) given by (5.10) and (5.11), respectively. The stable algebraic limit cycles, corresponding to $\delta _2=2$ and $\delta _4=4$, are plotted as the black lines, while the unstable algebraic limit cycles, corresponding to $\delta _1=1$ and $\delta _3=3$, are plotted as the green lines (color figure online)

Then the vector field $(f_0,g_0)$ has one critical point at $(x,y)=(1,1)$, which is inside the ovals (4.27) for any $\delta _i > 0.$ Consider $h_0(x,y)$ in the ODE system (4.29)–(4.30) in the product form (4.28) where $N=4$, $\delta _1=1$, $\delta _2=2$, $\delta _3=3$ and $\delta _4=4.$ Such curves have been visualized in Fig. 4a. They are algebraic limit cycles of the ODE system (4.29)–(4.30). In Fig. 6a, we plot ten illustrative trajectories of the ODE system (4.29)–(4.30). We observe that the trajectories starting at the corners of our visualized domain $[0,3.5] \times [0,3.5]$ approach the outer limit cycle corresponding to $\delta _4=4,$ while trajectories starting inside this oval converge either to it, or to the limit cycle corresponding to $\delta _2=2$ or to a fixed point. The limit cycles corresponding to $\delta _2=2$ and $\delta _4=4$ are stable and they satisfy our transversality condition (5.7). This is also confirmed in Fig. 6b, where we visualize the subdomains

5.10 $$\begin{aligned} \Omega _s&= \left\{ (x,y) \in [0,\infty )^2 \; \bigg | \; f_0(x,y) \, \frac{\partial h}{\partial x}(x,y) \, + \, g_0(x,y) \, \frac{\partial h}{\partial y}(x,y) \, < \, 0 \right\} \end{aligned}$$

5.11 $$\begin{aligned} \Omega _u&= \left\{ (x,y) \in [0,\infty )^2 \; \bigg | \; f_0(x,y) \, \frac{\partial h}{\partial x}(x,y) \, + \, g_0(x,y) \, \frac{\partial h}{\partial y}(x,y) \, > \, 0 \right\} \end{aligned}$$

using magenta and white shading, respectively. The limit cycles corresponding to $\delta _2=2$ and $\delta _4=4$ are inside the domain $\Omega _s$, while the limit cycles corresponding to $\delta _1=1$ and $\delta _3=3$ are inside the domain $\Omega _u$ and they are unstable, satisfying the transversality condition (5.8).

The systems with limit cycles which are used to achieve lower bounds in Tables 1 and 2 have been constructed using the standard definition of the limit cycle as an isolated closed trajectory. While such limit cycles can be stable, they are sometimes difficult to observe in numerical simulations. For example, consider the ODE system (4.7)–(4.8) which is a polynomial system of degree 4 with three hyperbolic algebraic limit cycles in the positive quadrant. The ODE system (4.7)–(4.8) shares some similarities with our general form (5.1)–(5.2) for $(f_0,g_0)=(1,1)$ if factor $\varepsilon {\hspace{0.56905pt}} x {\hspace{0.56905pt}} y$ is replaced by $7x-y.$ However, if we define the subdomains $\Omega _s$ and $\Omega _u$ by (5.10)–(5.11) for $(f_0,g_0)=(1,1)$, then we observe that some parts of each limit cycle of the ODE system (4.7)–(4.8) are in $\Omega _s$ and some parts are in $\Omega _u.$ While the application of Christopher (2001, Theorem 1) can help us to deduce that each limit cycle is hyperbolic, the trajectories are attracted by parts of the limit cycle in $\Omega _s$ and repelled by parts of the limit cycle in $\Omega _u$. In particular, numerical errors can make it impossible to observe a trajectory which would for long positive times (resp. long negative times) approach the (theoretically) stable (resp. unstable) limit cycle in computational studies of such systems. However, if we do not attempt to minimize the degree of the polynomial on the right hand side of the ODE system (5.1)–(5.2), then it is possible to find $f_0$ and $g_0$ such that all limit cycles are fully in $\Omega _s$. We state this result as our next theorem.

### Theorem 3

Let $h: {{\mathbb {R}}}^2 \rightarrow {{\mathbb {R}}}$ be a polynomial of degree $n_h$ and let the real algebraic curve $h(x,y)=0$ contain $N \in {{\mathbb {N}}}$ ovals in the (strictly) positive quadrant $(0,\infty ) \times (0,\infty )$. Assume that

5.12 $$\begin{aligned} \nabla h(x,y) = \begin{pmatrix} \displaystyle \frac{\partial h}{\partial x}(x,y) \\ \displaystyle \frac{\partial h}{\partial y}(x,y) \end{pmatrix} \,\ne \, \begin{pmatrix} 0 \\ 0 \end{pmatrix} \qquad \text{ for } \text{ all }\, (x,y)\, \text{ satisfying }\, h(x,y)=0. \end{aligned}$$

Then the ODE system

5.13 $$\begin{aligned} \frac{\text{ d }x}{\text{ d }t}= &  - \, x \, y \, h(x,y) \, \frac{\partial h}{\partial x}(x,y) \, - \, \varepsilon \, x \, y \, \frac{\partial h}{\partial y}(x,y) , \end{aligned}$$

5.14 $$\begin{aligned} \frac{\text{ d }y}{\text{ d }t}= &  - \, x \, y \, h(x,y) \, \frac{\partial h}{\partial y}(x,y) \, + \, \varepsilon \, x \, y \, \frac{\partial h}{\partial x}(x,y) , \end{aligned}$$

is a polynomial ODE system of degree $n=2\,n_h+1$ which can be realized as a chemical reaction network under mass-action kinetics (for any value of parameter $\varepsilon )$. The chemical system (5.13)–(5.14) has *N* stable algebraic limit cycles contained in the components of the curve $h(x,y)=0$ for all $\varepsilon >0$ and the cofactor, defined by (4.1), is equal to $s(x,y) \,=\, - \, x \, y \parallel \! \nabla h(x,y) \!\parallel ^2.$

### Proof

Consider the ODE system (5.1)–(5.2) with

5.15 $$\begin{aligned} f_0(x,y) \, = \, - \, x \, y \, \frac{\partial h}{\partial x}(x,y) \qquad \text{ and } \qquad g_0(x,y) \, = \, - \, x \, y \, \frac{\partial h}{\partial y}(x,y). \end{aligned}$$

Then the ODE system (5.1)–(5.2) becomes the ODE system (5.13)–(5.14), where the right-hand side contains polynomials of degree at most $2 n_h + 1.$ Moreover, the assumption (5.12) implies the transversality condition (5.7). Using Theorem 2, we conclude the existence of *N* stable algebraic limit cycles contained in the components of the curve $h(x,y)=0$ for all $\varepsilon >0$. Differentiating *h*(*x*, *y*) with respect of time, we obtain

$$\begin{aligned} \frac{\text{ d }}{\text{ d }t} h(x,y) \,= &  \, \frac{\partial h}{\partial x}(x,y) \, \frac{\text{ d }x}{\text{ d }t} + \frac{\partial h}{\partial y}(x,y) \, \frac{\text{ d }y}{\text{ d }t} \,\\= &  \, - \, x \, y \, \left[ \left( \frac{\partial h}{\partial x}(x,y) \right) ^{\!\!2} + \left( \frac{\partial h}{\partial y}(x,y) \right) ^{\!\!2} \right] h(x,y) \end{aligned}$$

which implies that the cofactor (4.1) is a polynomial of degree at most $2 n_h$ given by

$$\begin{aligned} s(x,y) \,=\, - \, x \, y \, \left( \frac{\partial h}{\partial x}(x,y) \right) ^{\!\!2} - \, x \, y \, \left( \frac{\partial h}{\partial y}(x,y) \right) ^{\!\!2} \,=\, - \, x \, y \parallel \! \nabla h(x,y) \!\parallel ^2. \end{aligned}$$

$$\square $$

We note that the ODE system (5.13)–(5.14) can also be written in the matrix form as

5.16 $$\begin{aligned} \frac{\text{ d }}{\text{ d }t} \begin{pmatrix} x \\ y \end{pmatrix} \, = \, x \, y \, \begin{pmatrix} -h(x,y) & - \varepsilon \\ \varepsilon & -h(x,y) \end{pmatrix} \nabla h(x,y) . \end{aligned}$$

This ODE system can be used to construct chemical systems with multiple stable algebraic limit cycles, provided that the ovals of $h(x,y)=0$ are contained in the (strictly) positive quadrant $(0,\infty )^2$, as we illustrate using examples with quartic planar curves (i.e. using $n_h=4$) in the next section.

### A chemical system with multiple stable algebraic limit cycles

We consider quartic polynomial *q*(*x*, *y*) in the following form

**Fig. 7:**
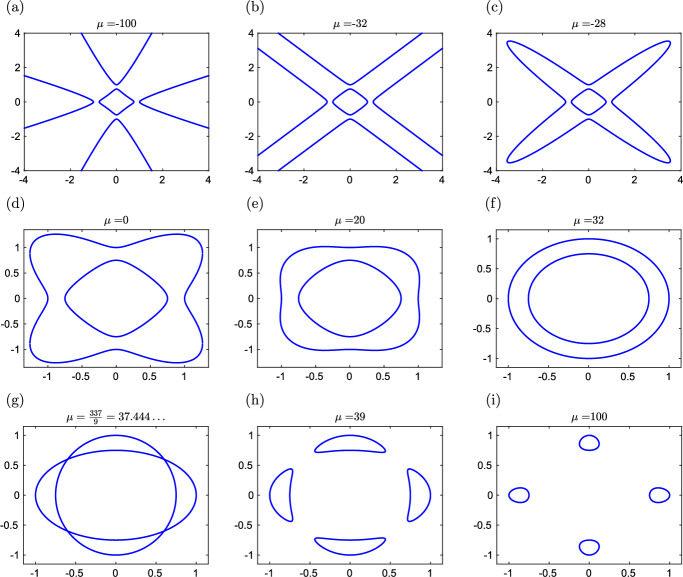
The algebraic curve $q(x,y)=0$ given by (5.17) for (**a**) $\mu =-100$; (**b**) $\mu =-32$; (**c**) $\mu =-28$; (**d**) $\mu =0$; (**e**) $\mu =20$; (**f**) $\mu =32$, (**g**) $\mu =337/9$; (**h**) $\mu =39$ and (**i**) $\mu =100$

5.17 $$\begin{aligned} q(x,y) \,= \, 16 \, (x^4+y^4) \, - \, 25 \, (x^2+y^2) \, + \, \mu \, x^2 y^2 \, + \, 9 , \end{aligned}$$

where $\mu \in {{\mathbb {R}}}$ is a parameter. Since the degree of the polynomial (5.17) is 4 for all $\mu \in {{\mathbb {R}}}$, Harnack’s curve theorem implies that the maximum number of connected components of the algebraic curve $q(x,y)=0$ is 4. Depending on the value of parameter $\mu \in {{\mathbb {R}}},$ the algebraic curve $q(x,y)=0$ contains one, two or four ovals, as we show in our next lemma and illustrate in Fig. 7.

#### Lemma 13

Let $\mu \in {{\mathbb {R}}}$ and let *q*(*x*, *y*) be given by (5.17). Then we have:

(i) The set of solutions to equation $q(x,y)=0$ contains points
  5.18 $$\begin{aligned} &  {[}-1,0], \quad [-3/4,0], \quad [3/4,0], \quad [1,0], \nonumber \\ &  {[}0,-1], \quad [0,-3/4], \quad [0, 3/4], \quad \text{ and } \quad [0,1]. \end{aligned}$$
  Points (5.18) are the only intersections of the algebraic curve $q(x,y)=0$ with *x*-axis and *y*-axis.
(ii) If $\mu \le -32,$ then the set of solutions to equation $q(x,y)=0$ contains one oval.
(iii) If $-32< \mu < 337/9,$ then the set of solutions to equation $q(x,y)=0$ contains two ovals.
(iv) If $\mu = 32,$ then the algebraic curve $q(x,y)=0$ are two concentric circles with radii 3/4 and 1.
(v) If $\mu =337/9$, then the set of solutions to equation $q(x,y)=0$ is connected and contains four ordinary double points (crunodes) at [3/5, 3/5], $[3/5,-3/5]$, $[-3/5,3/5]$ and $[-3/5,-3/5]$.
(vi) If $\mu > 337/9,$ then the set of solutions to equation $q(x,y)=0$ contains four ovals.

In particular, $q(x,y)=0$ is an M-curve containing four connected components.

#### Proof

(i) If $y=0$, then $q(x,y)=0$ simplifies to $16 x^4 \, - \, 25 x^2 \, + \, 9 \, = \, 0,$ which is solved by $x=\pm 1$ and $x=\pm 3/4.$ Using symmetry, equation $q(x,y)=0$ is solved for $x=0$ by $y=\pm 1$ and $y=\pm 3/4.$

(ii) Using (5.17), we have

$$\begin{aligned} q(x,x) \,= \, (32+\mu ) \, x^4 \, - \, 50 \, x^2 \, + \, 9 . \end{aligned}$$

If $\mu \le -32$, then equation $q(x,x)=0$ has exactly two real solutions and the algebraic curve $q(x,y)=0$ contains one oval. For example, if $\mu =-32$, then the two real solutions to $q(x,x)=0$ are given as $x=\pm 3/(5\sqrt{2}) \approx 0.424$ and the algebraic curve $q(x,y)=0$ contains one oval which goes clockwise through the points $[-3/4,0],$ $[-3/(5\sqrt{2}),3/(5\sqrt{2})],$ [0, 3/4],  $[3/(5\sqrt{2}),3/(5\sqrt{2})],$ [3/4, 0],  $[3/(5\sqrt{2}),-3/(5\sqrt{2})],$ $[0,-3/4]$ and $[-3/(5\sqrt{2}),3/(5\sqrt{2})],$ see Fig. 7b.

(iii) If $-32< \mu < 337/9,$ then there are four real solutions to $q(x,x)=0$ given by

$$\begin{aligned} \pm \sqrt{\frac{25 + \sqrt{337 - 9 \, \mu }}{ 32+\mu }} \quad \text{ and } \quad \pm \sqrt{\frac{25 - \sqrt{337 - 9 \, \mu }}{32+\mu }} \end{aligned}$$

and the set of solutions to equation $q(x,y)=0$ contains two concentric ovals, see Fig. 7c–f.

(iv) If $\mu =32$, then the formula (5.17) can be rewritten as

$$\begin{aligned} q(x,y) \,= \, 16 \, (x^2+y^2)^2 \, - \, 25 \, (x^2+y^2) \, + \, 9 \,=\, 16 \, r^4 - \, 25 \, r^2 + \, 9 , \end{aligned}$$

where $r^2=x^2+y^2.$ Solving $q(x,y)=0$ for *r*, we obtain $r=1$ or $r=3/4,$ see Fig. 7f.

(v) If $\mu =337/9$, then there are two solutions to $q(x,x)=0$ given as $\pm 3/5.$ They correspond to ordinary double points (crunodes) at [3/5, 3/5], $[3/5,-3/5]$, $[-3/5,3/5]$ and $[-3/5,-3/5]$, where the curves intersects itself so that two branches of the curve have distinct tangent lines, see Fig. 7g.

(vi) If $\mu > 337/9,$ then there are no solutions to $q(x,x)=0$. In particular, we have four regions separated by lines $y=x$ and $y=-x$ each containing one oval, see Fig. 7h and i. $\square$

The ovals of the algebraic curve *q*(*x*, *y*) in Lemma 13 are outside of the positive quadrant. To apply Theorem 3, we first shift the curve *q*(*x*, *y*) to get

5.19 $$\begin{aligned} h(x,y) = q(x-2,y-2), \qquad \text{ where }\, q(x,y)\,\text{ is } \text{ given } \text{ by }\, (5.17). \end{aligned}$$

**Fig. 8:**
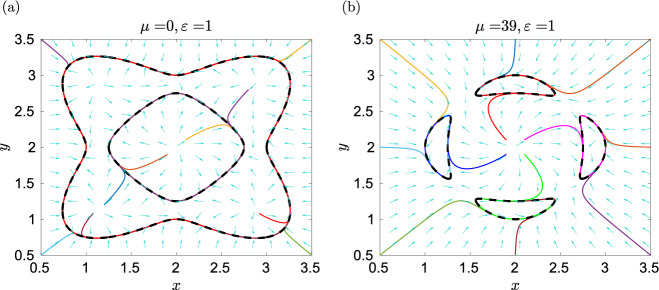
(**a**) The phase plane of the ODE system (5.13)–(5.14) with the polynomial *h*(*x*, *y*) given by (5.19) for parameters $\mu =0$ and $\varepsilon =1.$ We plot the two ovals of the algebraic curve $h(x,y)=0$ (black dashed line) together with ten illustrative trajectories showing convergence to one of the two ovals which are stable algebraic limit cycles of the ODE system. (**b**) The phase plane of the ODE system (5.13)–(5.14) with the polynomial *h*(*x*, *y*) given by (5.19) for parameters $\mu =39$ and $\varepsilon =1,$ when the ODE system has four stable algebraic limit cycles given as the four ovals of the algebraic curve $h(x,y)=0$ (visualized as the black dashed line together with illustrative trajectories converging to the limit cycles)

Then the ovals of *h*(*x*, *y*) are in the positive quadrant for all nonnegative values of $\mu ,$ see Fig. 7. The phase plane of the ODE system (5.13)–(5.14) is plotted in Fig. 8 for $\varepsilon =1$. We use two different values of $\mu$ corresponding to two ovals ($\mu =0$) and four ovals ($\mu =39$) of the algebraic curve $h(x,y)=0$ given by (5.19). In both cases, we observe that all computed illustrative trajectories approach one of the ovals, confirming that Theorem 3 leads to chemical systems with two (Fig. 8a) or four (Fig. 8b) stable limit cycles.

## Discussion

We have considered both algebraic and non-algebraic limit cycles in chemical reaction systems, with our results summarized together with the results in the literature in Tables 1 and 2, respectively. To establish some lower bounds in Tables 1 and 2, different techniques have to be applied. For example, small perturbations of an ODE system preserve the existence of a hyperbolic limit cycle, but an algebraic limit cycle can become non-algebraic after a perturbation. In particular, while the existence of a cubic weakly reversible chemical system with a limit cycle has been established in Table 1, it remains an open question whether a cubic weakly reversible system can have an algebraic limit cycle.

While a formulation of Hilbert’s 16th problem restricted to algebraic limit cycles under generic conditions has been solved, see Llibre et al. (2010); Giné et al. (2018) for further discussion, these results are not considering the ODE systems which can be realized as chemical reaction networks. For example, a cubic system with two circular limit cycles is presented in Giné et al. (2018). Shifting the limit cycles to the positive quadrant, as we have done with our quartic example in equation (5.19), and then multiplying the right-hand-side by *xy* yields a fifth-order chemical system with two limit cycles. Other examples of cubic systems with 2 (non-generic) algebraic limit cycles appear in Llibre et al., (2010, Section 1), which could again be used to conclude that $S^a(5)\ge 2$. However, this does not improve the lower bound in Table 2, which implies $S^a(5) \ge S^a(4) \ge 3.$

Our investigation has focused on the ODE systems which can be realized as models of chemical reaction networks. However, such a realization is not unique: if an ODE system can be realized as the reaction rate equations of a chemical system, then there exists infinitely many chemical reaction networks corresponding to the same ODE system (Craciun and Pantea 2008; Plesa et al. 2018; Craciun et al. 2020). For some studied ODE systems, we have been able to identify their realization as (weakly) reversible chemical reaction networks and this helped us to make conclusions on the values of numbers *W*(*n*) and $W^a(n)$ (see, for example, our proof of Lemma 11). In particular, chemical reaction networks (corresponding to the same ODE system) can be distinguished by having different structural properties. They can also be distinguished by considering their more detailed stochastic description (Enciso et al. 2021), written as the continuous-time discrete-space Markov chain and simulated by the Gillespie algorithm (Gillespie 1977; Erban and Chapman 2020). While the long-term dynamics of some chemical reaction networks can consist of a unique attractor of their ODE models, the long-term (stationary) probability distribution given by their stochastic model may display multiple maxima (Duncan et al. 2015; Plesa et al. 2019). Considering chemical systems with limit cycles and oscillatory behaviour, stochastic models can bring additional possibilities for long-term dynamics including noise-induced oscillations (Muratov et al. 2005; Erban et al. 2009). It may also happen that the ODE has a periodic solution and the long-term probability distribution is degenerate, converging to the state with zero molecules of all chemical species as time $t \rightarrow \infty$ (Reddy 1975).

The ODE system (5.13)–(5.14) or in its equivalent matrix form (5.16) can be used to construct chemical reaction networks with stable algebraic limit cycles corresponding to the given algebraic curve $h(x,y)=0.$ In particular, if we want to construct a chemical system with more than one stable algebraic limit cycle, we can start with a quartic curve with more than one oval as shown in Sect. 5.1. Another quartic curve with two ovals can be obtained as a product of two circles (quadratic curves). Such a product form construction has been used in our proof of Theorem 1, see equation (4.28). Considering the product of two circles and using Theorem 3, we can obtain a chemical system which has the two circles as its two stable algebraic limit cycles. In Sect. 5.1, we have considered quartic curve (5.19) which had four closed connected components for $\mu >337/9$ and Theorem 3 implied a chemical system with four stable algebraic limit cycles. To construct chemical systems with more stable limit cycles than four, we can apply Theorem 3 to algebraic curves $h(x,y)=0$ of degree $n_h>4,$ which has the corresponding number of ovals. One possible way to find such algebraic curves is to construct them in the product form (4.28).

In this paper, we have considered chemical reaction systems with two chemical species *X* and *Y* which are described by planar ODE system (1.1)–(1.2). In particular, we could make connections to the results and open problems on limit cycles and periodic solutions in planar polynomial ODE systems, with attention to the results for systems with polynomials of low degree *n* on the right hand side (Shi 1980; Li et al. 2009). Our low degree *n* investigation is also interesting from the applications point of view, because it decreases the order (2.2) of the chemical reactions when the ODE system (1.1)–(1.2) is realized as the chemical system. In particular, we have addressed some questions on ‘minimal’ reaction systems with certain dynamics by minimizing the value of *n*. The minimal reaction systems with oscillations can also be defined in terms of the minimal number *m* of reactions in the chemical reaction network (2.1), see Banaji et al. (2024) for some systems with two chemical species. In some applications, it is necessary to study chemical reaction systems with more than two chemical species, leading to three-dimensional or higher-dimensional ODE systems. For example, limit cycles in reaction networks with three or four chemical species are investigated under additional structural conditions on the reaction network in Boros and Hofbauer (2022, 2023). Multiple limit cycles for systems of two chemical species have also been reported in Boros and Hofbauer (2024) for the case when deficiency of the chemical reaction network is one, while it is well known that the deficiency-zero networks cannot have periodic solutions in the positive quadrant (Feinberg 1972).

## Data Availability

The manuscript contains no data.
